# Fluorescent blood–brain barrier tracing shows intact leptin transport in obese mice

**DOI:** 10.1038/s41366-018-0221-z

**Published:** 2018-10-03

**Authors:** Luke Harrison, Sonja C. Schriever, Annette Feuchtinger, Eleni Kyriakou, Peter Baumann, Katrin Pfuhlmann, Ana C. Messias, Axel Walch, Matthias H. Tschöp, Paul T. Pfluger

**Affiliations:** 1 Research Unit Neurobiology of Diabetes, Helmholtz Zentrum München, 85764 Neuherberg, Germany; 2 Institute for Diabetes and Obesity, Helmholtz Zentrum München, 85764 Neuherberg, Germany; 3 German Center for Diabetes Research (DZD), 85764 Neuherberg, Germany; 4 Division of Metabolic Diseases, Technische Universität München, 80333 Munich, Germany; 5 Research Unit Analytical Pathology, Helmholtz Zentrum München, 85764 Neuherberg, Germany; 6 Institute of Structural Biology, Helmholtz Zentrum München, 85764 Neuherberg, Germany; 7 Biomolecular NMR and Center for Integrated Protein Science Munich at Department Chemistry, Technical University Munich, 85747 Garching, Germany

**Keywords:** Obesity, Hypothalamus

## Abstract

**Background/objectives:**

Individuals carrying loss-of-function gene mutations for the adipocyte hormone leptin are morbidly obese, but respond favorably to replacement therapy. Recombinant leptin is however largely ineffective for the vast majority of obese individuals due to leptin resistance. One theory underlying leptin resistance is impaired leptin transport across the blood–brain-barrier (BBB). Here, we aim to gain new insights into the mechanisms of leptin BBB transport, and its role in leptin resistance.

**Methods:**

We developed a novel tool for visualizing leptin transport using infrared fluorescently labeled leptin, combined with tissue clearing and light-sheet fluorescence microscopy. We corroborated these data using western blotting.

**Results:**

Using 3D whole brain imaging, we display comparable leptin accumulation in circumventricular organs of lean and obese mice, predominantly in the choroid plexus (CP). Protein quantification revealed comparable leptin levels in microdissected mediobasal hypothalami (MBH) of lean and obese mice (*p* = 0.99). We further found increased leptin receptor expression in the CP (*p* = 0.025, *p* = 0.0002) and a trend toward elevated leptin protein levels in the MBH (*p* = 0.17, *p* = 0.078) of obese mice undergoing weight loss interventions by calorie restriction or exendin-4 treatment.

**Conclusions:**

Overall, our findings suggest a crucial role for the CP in controlling the transport of leptin into the cerebrospinal fluid and from there to target areas such as the MBH, potentially mediated via the leptin receptor. Similar leptin levels in circumventricular organs and the MBH of lean and obese mice further suggest intact leptin BBB transport in leptin resistant mice.

## Introduction

Maintenance of energy homeostasis is the keystone in preventing obesity and the metabolic syndrome. The brain plays an important role in orchestrating this homeostasis by receiving inputs from peripheral tissues and responding by regulating various sensations such as reward and satiety. In addition to this, direct innervation as well as indirect signaling via hormones allows for a feedback system back to the peripheral organs [1]. The transport of these peripheral signals into the brain and the target regions is regulated in a controlled manner by the blood–brain barrier, in essence a layer of endothelial cells that divides the microvasculature from the brain compartment. Several cell types such as pericytes and astrocytes contribute to the architecture and function of the blood–brain barrier (BBB), which ultimately protects the brain from neurotoxins while governing the passive diffusion of gases and hydrophobic molecules as well as the active transport of hydrophilic nutrients, amino acids and large-scale peptide hormones [2, 3].

One such peptide hormone which requires active BBB transport is leptin, a 16 kDa hormone that is synthesized in white adipose tissue and released into circulation in direct proportion to the amount of body fat [4, 5]. Leptin serves as an adipostat, i.e., it has the capacity to inform the rest of the body on available fat stores. The importance of leptin in the control of systemic energy homeostasis is demonstrated by clinical observations in people with genetic loss of leptin, which develop hyperphagia and morbid obesity in early childhood that can be reversed by leptin replacement therapy [6]. Most obese subjects are however not deficient in leptin, but rather leptin resistant. Despite high circulating leptin levels, leptin resistant individuals typically experience a drive to eat extra calories, which impedes sustainable weight loss [7]. Molecular reasons for leptin resistance are not yet entirely clear, but may entail impaired signaling linked to SOCS3 expression [8], elevated levels of circulating c-reactive protein [9] or impaired histone deacetylase 5 activity in the hypothalamus [10]. Moreover, impaired transport of leptin into the brain is viewed as important contributor to leptin resistance [11]. This is mainly supported by leptin transport studies that used radioactively labeled leptin and showed a decrease in blood–CSF leptin ratios, suggesting blunted leptin transport kinetics and thus a cause for leptin resistance [12]. Similarly, El-Haschimi and colleagues performed a series of intracerebral ventricular (ICV) leptin injection experiments, which also indicated a decrease in leptin BBB transport [13].

Leptin must cross the BBB to reach its neuronal targets in the mediobasal hypothalamus (MBH) and other brain areas. How leptin is transported across the BBB and to its responder neurons, defined by their expression of the leptin receptor (LepR), is still a matter of debate [14]. Past work hints that leptin BBB transport is facilitated by tanycytes, i.e., specialized ependymal cells in the median eminence (ME), a circumventricular organ at the bottom of the MBH involved in secreting brain derived signals to the pituitary and peripheral organs via the circulation [15, 16]. In the ME, leptin can freely diffuse into the ME parenchyma due to the fenestration of capillaries and lack of a functional BBB. Subsequently, leptin is transported via tanycytes into the cerebrospinal fluid (CSF) of the ventricular space, from where it then laterally diffuses into the MBH [17]. More recent work points towards a crucial direct involvement of endothelial cells of the BBB as major mechanism of leptin transport [18]. The knockdown of LepR specifically in endothelial cells of the BBB was functionally linked to impaired transport of leptin into the CSF and LepR positive brain regions, and aggravated obesity when mice were exposed to high fat diet [18]. Dense LepR expression is especially found in the choroid plexus (CP) [19], an important component of the BBB anchored to the walls of the lateral, central and fourth ventricles. Although the production of CSF is the most described role of the CP, it also acts as an important selective gateway to the CSF. Molecules that enter the CSF can freely diffuse into many brain regions that line the ventricles [20]. Accordingly, the CP has been strongly linked to leptin transport into the brain [21].

Here, we aimed to gain new understanding in leptin BBB transport by visualizing and comparing leptin transport into the brain via whole mouse brain 3D imaging from lightsheet fluorescence microscopy. We furthermore aimed to address the question of whether leptin transport into the CP, ME and MBH is altered in diet-induced obese (DIO) and thus leptin resistant mice, compared to chow-fed and leptin sensitive lean control mice. Moreover, we compared leptin levels in the ME and MBH of DIO mice subjected to weight loss by either modest dieting, profound calorie restriction or repeated treatment with exendin-4, to clarify whether altered leptin transport into the MBH can explain the superior restoration of leptin sensitivity by pharmacology [22].

## Materials and methods

### Animals

All experiments were performed in adult male C57BL/6 J mice purchased from Janvier Labs (Saint-Berthevin, Cedex, France). Mice were maintained on a 12 h-light–dark cycle with free access to water and standard chow diet (Altromin, #1314) or 58% high fat diet (HFD) (Research Diets, D12331). Diet induced obese (DIO) mice were subjected to HFD for at least 20 weeks. Body composition was determined using nuclear magnetic resonance (NMR) technology (EchoMRI, Houston, TX, USA). For the diet intervention study, DIO mice were subdivided into 4 experimental groups. The group termed HFD was kept on HFD during the study. The remaining 3 groups of DIO mice were switched to chow on day 0 of the study and divided as follows: Diet switch (H > C) animals received ad libitum access to chow diet. Calorie restricted (CR) mice were restricted to the average food intake of the exendin-4 (EX4) group and EX4 treated animals were subjected to daily injections of exendin-4 (0.08 mg/kg) (Tocris biosciences, Bristol, UK) in the morning for up to 10 days. Age-matched mice fed chow were used as a control group. At the end of the diet-intervention study, all mice were first subjected to a single intraperitoneal (i.p.) injection of either vehicle or leptin, 45 min before being sacrificed by cervical dislocation for organ withdrawal. Brains were extracted swiftly and the ME and MBH dissected as described below. Recombinant murine leptin was reconstituted in 20 mM Tris-HCI, pH 8.0 at a concentration of 5 mg/ml. This was then further diluted in saline (0.9 % NaCl) to a final concentration of 1 mg/ml and injected at a dose of 5 mg/kg body weight. Mice were distributed into treatment groups based on their starting body weight. We thereby aimed to assure an equal distribution of starting body weights at the beginning of the study, which allows for better dissection of longitudinal treatments effects on body weight. In vivo experiments were performed without blinding of the investigators. All studies were based on power analyses to assure adequate sample sizes, and approved by the State of Bavaria, Germany.

### Leptin coupling

Leptin was coupled to either infrared IRDye® CW-800 (LICOR #929-71012) or far-red IRDye® 650 (LICOR #929-70020) fluorescent dyes. Coupling was carried out according to the IRDye® CW-800 kit handbook. Lyophilized recombinant mouse leptin (R&D systems Cat.# 498-OB-05M) was reconstituted in PBS with a pH of 8.5 to a final concentration of 1 mg/ml. The dye was reconstituted in RNAse-free water and the appropriate volume was added to 1 ml of 1 mg/ml leptin, then incubated at 20 °C for 2 h. Coupled leptin was separated from unbound dye by size exclusion column filtration (Pierce Zebra™ desalting spin columns, Life Technologies #89891). Dye solution not used for coupling was diluted to the same absorption value as that of the coupled leptin sample and used as a control. Largely avoiding freeze-thaw cycles, coupled leptin was stored in dark tubes and kept at 4 °C for short-term storage and −20 °C for long-term storage.

### Ion exchange purification

To separate coupled from uncoupled leptin, we performed Ion Exchange Chromatography (IEX) using a Resource Q anion-exchange column with 1 ml volume (RESOURCE^TM^ Q, GE Healthcare) at pH 8. The leptin-CW800 sample underwent a buffer exchange, by sequential concentration/dilution steps into 20 mM TRIS-HCl pH 8 (Buffer A). After loading the sample onto the column, the column was washed with 10 column volumes of Buffer A. Separation was then achieved by applying a salt gradient based on increasing ionic strength to 0.5 M NaCl (50% Buffer B; 20 mM TRIS-HCl buffer pH 8, 1 M NaCl) at a flow rate of 4 ml/min and eluent volume of 20 columns. Finally, the column was washed with 5 column volumes of 100% Buffer B. For each fraction the purity and molecular weight was assessed by a Pierce BCA protein assay kit (Thermo Fisher Scientific Inc., Rockford, IL, USA) and SDS-PAGE, as described below.

### Median eminence and mediobasal hypothalamus dissection

Under a dissecting microscope the ME can be seen as a thin structure following the sagittal plane, lying on top of the hypothalamus. It was possible to see the fenestrated blood vessels running along the ME. Using very fine forceps (Dumont, 5SPSF – Inox – B), and applying light pressure parallel to either side of the ME, the ME was pinched away from the hypothalamus. Removing the ME causes a break in the third ventricle wall, and a small amount of fluid was seen leaving the ventricle. This may serve as confirmation that the ME was removed. To remove the MBH the brain was cut with a scalpel coronally, directly through the center of the hypothalamus. The two brain halves are then laid flat, to expose the hypothalamus face upwards. If the ME dissection was done correctly, the MBH will have a flat surface. If too much tissue was removed, it will have a concave surface. Using fine forceps, two 45° cuts were made vertically on either side of the MBH. A third cut was then made horizontally below the MBH, allowing the MBH to be lifted from the brain. Tissues were snap frozen in liquid nitrogen and stored at −80 °C.

### Protein extraction and western blotting

As ME and MBH samples only provide a very small amount of tissue, samples from 2 mice were pooled to provide sufficient protein levels for detection. Tissue lysis buffer consisted of RIPA buffer (Thermo Fisher Scientific Inc., Rockford, IL, USA) with the addition of 1× phosphatase- and protease-inhibitors (Thermo Fisher Scientific Inc., Rockford, IL, USA) and 1 mM phenyl-methane-sulfonyl fluorid (PMSF). Using 200 µl of lysis buffer for MBH samples and 50 µl of lysis buffer for ME samples resulted in the most efficient protein extraction. Samples were lysed via sonication then rotated on a wheel for 30 min to ensure full suspension of the tissues in lysis buffer. Samples were then centrifuged at 12,000×*g* for 7 min and the supernatants collected. Protein concentrations were measured using the Pierce BCA protein assay kit (Thermo Fisher Scientific Inc., Rockford, IL, USA), samples were then diluted to equal concentrations in 4x NuPage buffer + DTT (Thermo Fisher Scientific Inc., Rockford, IL, USA). After boiling at 95 °C for 5 min, equal amounts of protein were loaded onto 4–20% gradient Criterion™ TGX™ Precast Gels (Biorad, Hercules, CA, USA). Samples were transferred to a nitrocellulose membrane using the Trans-Blot® Turbo™ Transfer System (Biorad, Hercules, CA, USA). Membranes were blocked in Tris-buffered-saline with 0.05% Tween 20 (TBS-T) containing 5% BSA (VWR, Vienna, Austria) for 1 h. For leptin detection, membranes were blocked in 5% skim milk powder (Sigma-Aldrich, St. Louis, Missouri, USA) in TBS-T. Primary antibodies were anti-pSTAT3^T705^ (rabbit polyclonal, 1:1000, Cat #9145), anti-STAT3 (mouse monoclonal, 1:1000, Cat #9139), anti-β-actin (rabbit polyclonal, 1:10000, Cat #4970) (all antibodies purchased from Cell Signaling Technology (Cell Signaling, Danvers, MA, USA)) or anti-murine-leptin (rabbit polyclonal, 1:1000, Cat #500-P68. Peprotech, Rocky Hill, NJ, USA). Antibodies were diluted in blocking buffer (5% skim milk powder for leptin antibodies, 5% BSA for all others) and incubated on the membranes overnight at 4 °C. Detection was achieved using ECL Clarity (Biorad, Hercules, California, USA) and exposure to high-sensitivity films (Amersham Hyperfilm ECL (GE Healthcare Bio-Sciences, Pittsburgh, PA, USA)). Densitometric analysis was performed using ImageJ 1.51 (NIH, Bethesda, Maryland, USA).

### Cell culture

HEK293 cells were cultured in a 6-well plate with low glucose DMEM, with 10% FBS and 1% Pen/Strep. Transfection of pCAG-2A-H2B Venus_mOBRb-HA was carried out using the FuGene® HD transfection reagent (Promega, Madison, WI, USA) as per the kit’s instructions with 1.5 µg of mLepRb plasmid DNA and 4.5 µl of transfection reagent. 48 h post transfection, cells were placed in starvation medium (DMEM, with 0.1% FBS) for 4 h prior to leptin stimulation. Recombinant murine leptin was added to the cells at a final concentration of 10 nM. Cells were stimulated for 30 min with vehicle, leptin, CW-800 dye or leptin-CW800, washed twice with ice cold PBS and then snap frozen at −80 °C. Cellular proteins were isolated by adding 100 µl RIPA buffer with inhibitors and PMSF, and further treated as described above.

### RNA extraction and qPCR

RNA was extracted from tissue using the NucleoSpin RNA isolation kit (Macherey-Nagel, Düren, Germany). Equal amounts of RNA were reverse transcribed to cDNA using the QuantiTect Reverse Transcription kit (Qiagen, Hilden, Germany). Gene expression was analyzed using TaqMan probes for LRP2 (Mm01328171_m1), murine leptin receptor (Mm00440181_m1), and Hprt (Mm01545399_m1) as the housekeeping gene with the respective TaqMan mastermix (Thermo Fischer Scientific, Inc., Rockford, IL USA). qPCRs were carried out using a ViiA™ 7 Real Time PCR System (Applied Biosystems). Gene expression was evaluated using the Δ-Δ Ct method.

### Tissue clearing and light-sheet fluorescence microscopy

The dissected brain was fixed in PaxGene (PreAnalytiX, Hombrechtikon, Switzerland) according to the manufacturer’s recommendations and thereafter underwent a chemical procedure of optical clearing as described before [23]. Afterwards cleared whole mouse brains were imaged on a light-sheet fluorescence microscope (UltraMicroscope II, LaVision BioTec, Bielefeld, Germany). In order to visualize the lectin-647 (Thermo Fisher Scientific Inc., Rockford, IL, USA, Cat# L32451) a bandpass filter set with an excitation range of 640/30 and emission range of 690/50 was used in combination with an additional filter set (excitation: 740/35; emission: 795/50) for detection of leptin-CW800 signals. 3D-reconstruction and rendering was performed using Imaris vers.7.3 (Bitplane, Concord, MA, USA). Volume analysis was performed using Arivis Vision4D (Arivis, Munich, Germany).

### Immunohistochemistry

Brains were extracted after cervical dislocation and incubated in 4% paraformaldehyde at 4 °C overnight. Brains were then transferred to 20% sucrose in 0.1 M tris-buffered-saline (TBS) for 24–48 h. Brains were frozen at −20 °C, mounted with OCT and cut coronally on a cryostat into 30 µm sections. Free-floating sections were subjected to pre-treatment with ice-cold 100% methanol for 10 min at −20 °C, then to blocking for 1 h at RT in a buffer containing 0.25% gelatin and 0.5% Triton X 100 in 1x TBS. Primary anti-pSTAT3^T705^ antibody was incubated with brain sections overnight at 4 °C. After 3 × 5 min washing steps with TBS, the secondary donkey anti-rabbit-568 antibody (1:500 in blocking buffer, Thermofischer Scientific, Cat # A10042) was incubated with brain slices for 1 h at RT. After washing, the sections were counterstained with DAPI (1:10000) and mounted on slides. Images were captured with a Leica TCS SP5 microscope. Stack and overlay pictures were created using ImageJ image analysis software (v. 1.51, NIH, Bethesda, Maryland, USA). Brain regions were defined with the use of the DAPI counterstaining and the Allen brain Atlas (http://mouse.brain-map.org/static/atlas).

### Statistical analyses

Statistical analyses were performed using GraphPad Prism (GraphPad Software, Inc. La Jolla, CA, USA). Two-tailed Student’s t-tests or One-Way ANOVA with Bonferroni’s post tests were used to compare differences between phenotypes. P-values lower than 0.05 were considered significant. Significances were indicated as follow: **p < 0.01, ***p < 0.001, ****p < 0.0001 or, groups with significantly different values were indicated as different characters and groups not significantly different from each other were indicated with the same characters. All results are presented as means +/− SEM. Gaussian distribution was analyzed with the D’Agostino-Pearson omnibus test.

## Results

### Fluorescently labeled leptin allows for visualization of leptin distribution in the whole mouse brain

**Fig. 1:**
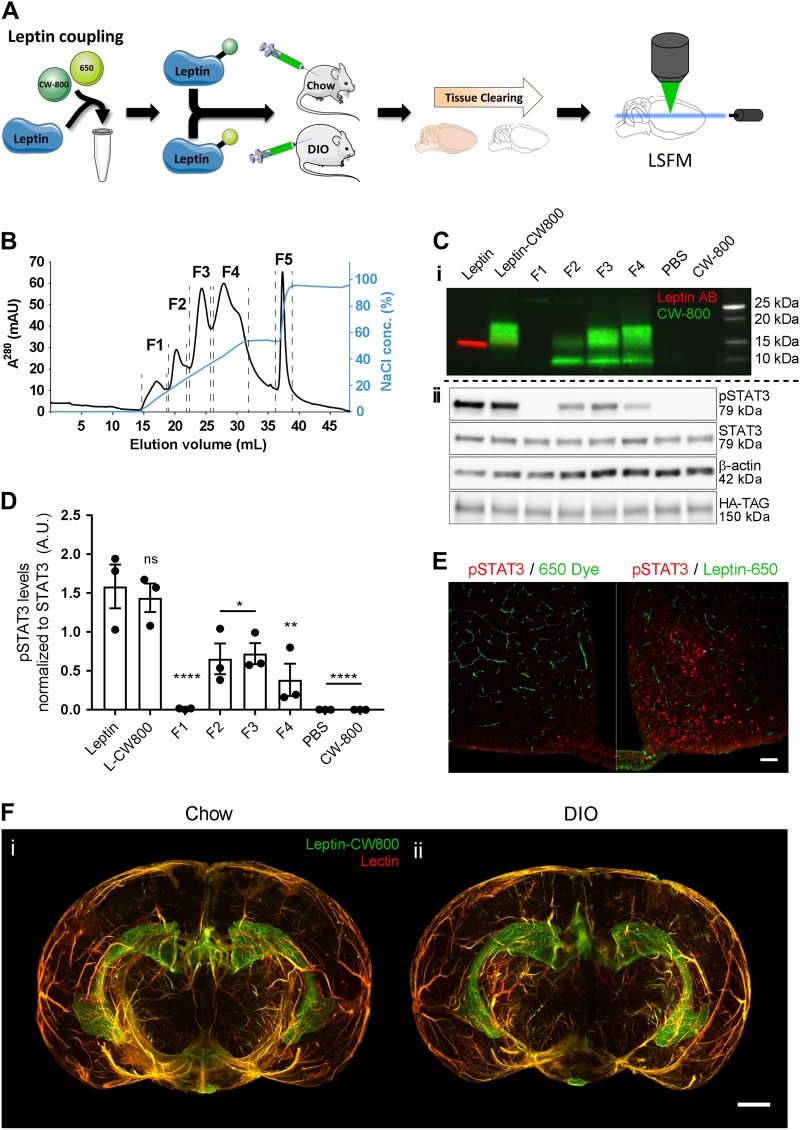
Labeled leptin combined with tissue clearing and light-sheet fluorescence microscopy allows for 3D visualization of leptin distribution in the intact mouse brain. **a** Leptin was coupled to either an infrared fluorescent dye (CW800) or a far-red fluorescent dye (650). Labeled leptin was then injected into chow-fed lean mice or age-matched diet-induced obese (DIO) mice fed with HFD for 20 weeks. Brains were collected 45 min after leptin injections and subjected to tissue clearing followed by LSFM to obtain 3D whole brain images. **b** Anion exchange chromatography was used to purify CW800 labeled leptin. Individual fractions F1–F4 were collected based on UV absorption (280 nm). A final fraction (F5) was collected by increasing the NaCl concentration above 50%, which is known to remove any remaining bound substances from the column. **c-i** Leptin, leptin-CW800, fractions F1–F4, PBS and CW800 alone were subjected to SDS-Page and Western blotting. Leptin bands were detected by either immunolabeling with a leptin antibody (red) or infrared fluorescence (green). **c-ii** Murine LepRb overexpressing HEK293 cells were incubated for 30 min with native leptin, leptin-CW800, fractions F1–F4, CW800 alone, or PBS to measure pSTAT3 levels as a marker for leptin bioactivity. **d** Densitometric analysis of the pSTAT3 signal from the western blots seen in **c-ii** . **e** Bioactivity was further confirmed in vivo by injecting leptin-650 (i.p., 5 mg kg^−1^) in mice and analyzing pSTAT3 45 min after leptin administration (pSTAT3 shown in red, leptin-650 or the 650 dye alone shown in green). **f** Mice, either chow-fed and lean (**i**) or HFD-fed and DIO (**ii**), were injected with leptin-CW800 (i.p., 5 mg kg^−1^) and lectin-647 (i.v., 250 µg). 3D-reconstruction of the brains reveals leptin accumulation in the ME and CP (Lectin-647 shown in red, leptin-CW800 shown in green). Scale bars for **e** and **f** are 100 and 1000 µm, respectively. Data in D are means ± SEM for 3 independent experiments. Significance was determined using One-Way ANOVA and Bonferonni’s post-hoc testing. Significance is depicted as *p < 0.05, **p < 0.01, ****p < 0.0001

To visualize how exogenous leptin distributes in the brain after injection (i.p., 5 mg kg^−1^), we coupled an infrared fluorescent dye (CW800) or a far-red fluorescent dye (650) to recombinant murine leptin (Fig. 1a). The product leptin-CW800 was subsequently subjected to anion exchange chromatography (Fig. 1b), and the resulting fractions 1 to 4, washout fraction 5 as well as the naïve unlabeled leptin and the labeled product leptin-CW800 were assessed by SDS page and Western Blotting (Fig. 1c). Immunolabeling with an antibody against leptin, and the simultaneous direct detection of infrared CW800 fluorescence revealed a full conversion of unlabeled leptin to leptin-CW800, i.e., the absence of unlabeled leptin in the reaction product (Fig. 1c–i). Moreover, fractions 2 to 4 contained leptin-CW800 bands that appeared to be larger in size (ca. 17–20 kDa) compared to naïve unlabeled leptin at 16 kDa. Notably, the antibody against leptin had a profoundly reduced affinity against leptin-CW800 (Fig. 1c–i), and leptin-CW800 could only be visualized when the signal intensity was strongly enhanced (Supplementary Figure 1A).

Next, to test if labeled leptin-CW800 was still bioactive, we overexpressed the long, signal transducing form of the murine leptin receptor (mLepRb) in HEK293 cells and stimulated these cells with either native leptin, leptin-CW800, the isolated fractions, vehicle (PBS) or CW800-dye alone. Labeled leptin-CW800 was capable of activating LepRb downstream signaling to the same extent as native leptin, indicated by comparable phosphorylation of signal transducer and activator of transcription 3 (STAT3, pSTAT3) (Fig. 1c-ii and 1d). Fractions 2–4 displayed significant leptin bioactivity as well, but proteolytic cleavage with the occurrence of a 10 kDa leptin fragment during anion exchange chromatography slightly diminished their bioactivity compared to native or labeled leptin-CW800. Accordingly, to confirm the bioactivity of labeled leptin in vivo, we injected 5 mg kg^−1^ of unfractionated leptin-650 into chow fed control mice and confirmed an increased staining for pSTAT3 nuclei in the hypothalamus compared to injection of the 650 dye alone (Fig. 1e). Having established that labeled leptin is functional in vitro and in vivo, we next injected unfractionated leptin-CW800 (i.p., 5 mg kg^−1^, 45 min prior to sacrifice) into mice fed either chow or a high fat diet (HFD). We also injected fluorescent lectin-647 (i.v., 250 µg, 5–10 min prior to sacrifice), which marks blood vessels by binding glycoproteins on the vascular endothelial layer [24].

**Fig. 2:**
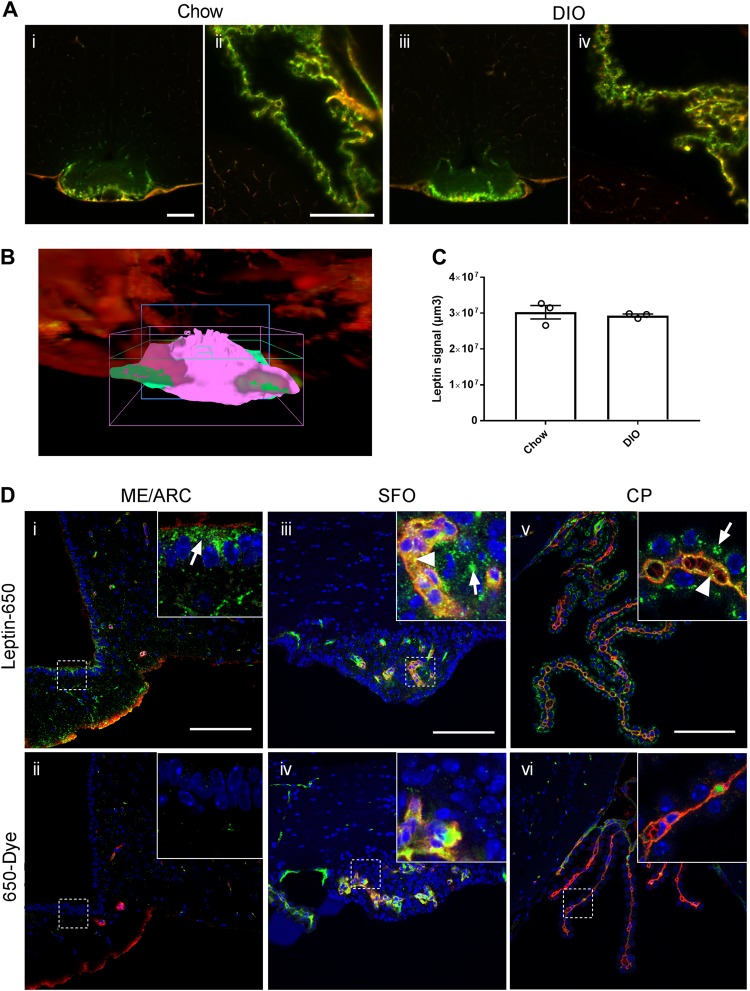
Accumulation of labeled leptin in circumventricular organs and the choroid plexus of mice. **a** Leptin-CW800 (i.p., 5 mg kg^−1^) and lectin-647 (i.v., 250 µg) were injected into either DIO or chow control mice, and the brains examined with LSFM. (Leptin-CW800 shown in green, lectin-647 shown in red). Leptin-CW800 that has entered the brain parenchyma is seen as green, whereas leptin-CW800 within the blood vessels is visualized as yellow. **a-i** and **a-iii** depict the ME and **a-ii** and **a-iv** depict the CP in the lateral ventricle. **b** Screenshot depicting the leptin volume (pink) and blood vessel volume (turquoise) calculations from the ARIVIS image analysis software package. **c** Quantification of the total leptin volume within the ME for lean chow fed and DIO mice. **d** To obtain higher resolution of specific regions, leptin-650 (i.p., 5 mg kg^−1^) or 650-Dye and lectin-488 (i.v., 250 µg) were injected into chow fed mice and examined via confocal microscopy. Arrows indicate leptin-650 in the brain parenchyma, arrowheads indicate leptin-650 within blood vessels. ME median eminence, ARC arcuate nucleus, SFO subfornical organ, CP choroid plexus (lateral ventricle). Scale bars for **a-i** and **ii** are 100 µm, and for **di–vi** 100 µm. Leptin-CW800/leptin-650 are shown in green, lectin-647/lectin488 are shown in red, DAPI is shown in blue. Data in **c** are means ± SEM for an *n* of 3. Significance was determined using a two-tailed student’s *t*-test

Tissue clearing and light-sheet fluorescence microscopy (LSFM) (Fig. 1a) allowed us to visualize precisely where exogenous leptin-CW800 accumulates in the brain (Fig. 1f). Leptin-CW800 that has entered the brain parenchyma is clearly distinct from leptin that is in circulation, which co-localizes with lectin in the blood vessels (yellow/orange signal) (Figs. 1f & 2a). 3D rendering of LSFM images allowed for further appreciation of the leptin-CW800 distribution in the brain. Using 3D visualization we could demonstrate, based on signal strength and overall size, that the majority of leptin-CW800 is seen in the lateral CP (Supplementary Video SV1, SV2 and SV5). Leptin-CW800 is predominantly found in the circumventricular organs such as the ME or the CP in the lateral ventricle (Fig. 2a). In 3D rendered LSFM images (Supplementary Video SV2), other circumventricular organs such as the CP in the 4th ventricle also showed high leptin-CW800 signal. In contrast, the arcuate nucleus (ARC) appears to be protected from circulating molecules that leak into the ME, evidenced by the restricted signal of leptin-CW800 within the ME and a lack of penetration into the ARC 45 min post leptin i.p. injection (Fig. 2a-i & ii). In the lateral ventricle CP the leptin signal was not only present in the blood vessels, but also outside the capillaries within the CP ependymal cell layer (Fig. 2a-ii, iv & Supplementary Video SV5). When compared to other microvessels of the brain, leptin-CW800 signal is found only colocalized within lectin-647 (Supplementary Video SV5). Interestingly, when comparing leptin-CW800 accumulation in chow and DIO mice, there appears to be no difference in signal intensities across all areas positive for leptin-CW800. DIO mice display no differences in leptin accumulation in the ME or CP ependymal layers (Fig. 1f, Fig. 2a, Supplementary Video SV1, SV3 and SV4). This was quantified using volumetric calculations based on signal intensity for the ME (Fig. 2b) and showed no difference between lean chow fed and HFD fed DIO mice (Fig. 2c).

Due to resolution limitations of the LSFM setup, it was not possible to distinguish whether or not the leptin-CW800 signal was in the CP interstitium, or had been taken up by the ependymal cells themselves. The latter would indicate a specific uptake, due to the fact that the ependymal cells are connected by tight-junctions and thus form the blood–CSF barrier in the CP. To overcome this we injected mice with leptin-650 (i.p., 5 mg kg^−1^) and lectin-488 (i.v., 250 µg), thereby enabling visualization by confocal microscopy (Fig. 2d). Here, we see that leptin-650 (Fig. 2d–i) but not dye alone (Fig. 2d–ii) is found to accumulate along the border of the ME to the third ventricle and within the subfornical organ (SFO) (Fig. 2d–iii, iv), which is another circumventricular organ that lacks an intact BBB. Furthermore, leptin-650 appears to be specifically taken up within the ependymal cells of the CP (Fig. 2d–v arrows), when compared to the dye injected alone (Fig. 2d–vi).

### Leptin downstream signaling in the MBH peaks 45 min after exogenous leptin administration

**Fig. 3:**
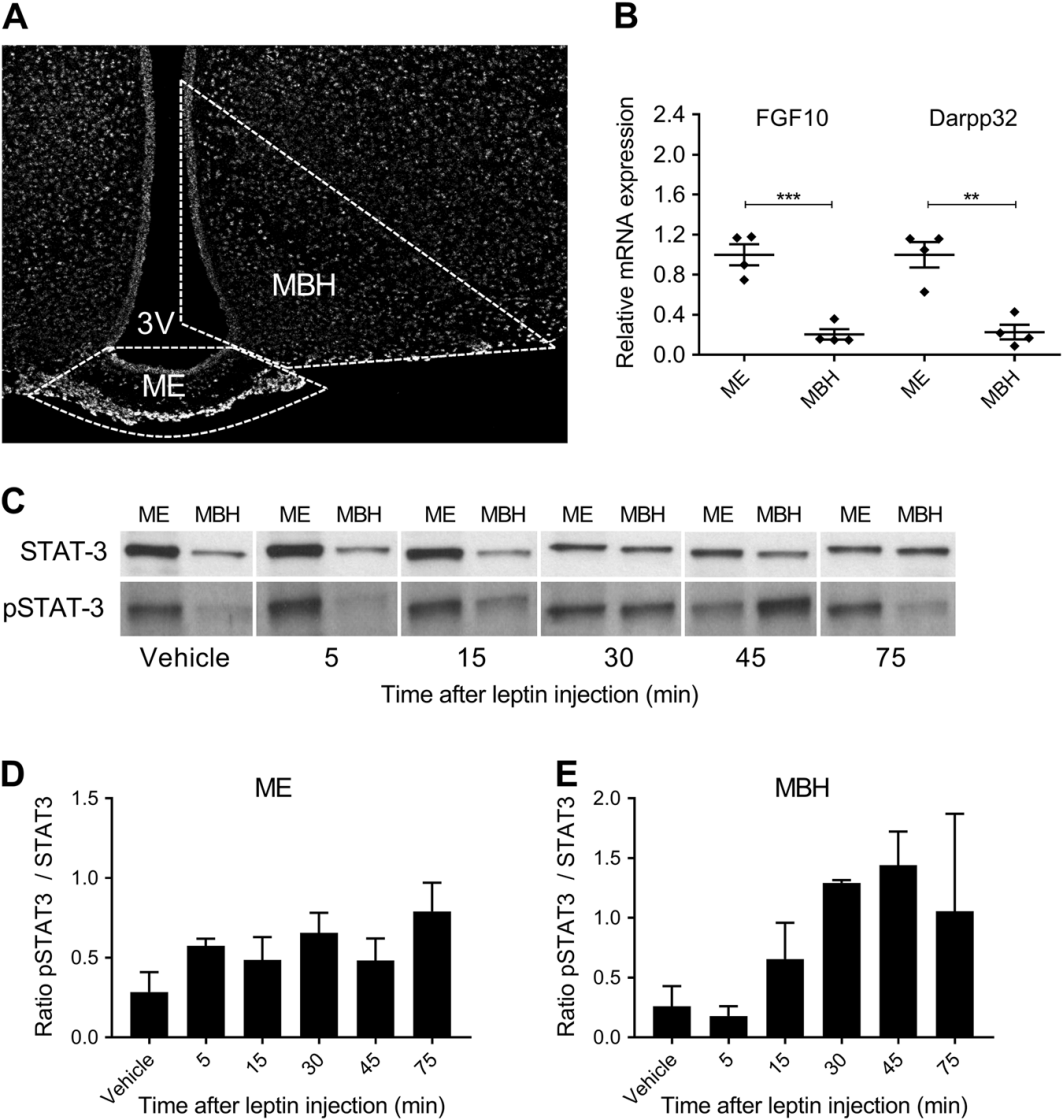
Leptin signaling in dissected median eminence (ME) and mediobasal hypothalamus (MBH) mouse brain tissue. **a** The ME and MBH were dissected from murine brains as depicted in **a**. **b** A correct dissection was determined using RT-qPCR of specific ME-residing tanycyte genes (FGF10 and Darpp32). **c**–**e** Leptin transport kinetics into the ME and MBH were assessed in mice injected with leptin (i.p., 5 mg kg^−1^) or vehicle. ME and MBH tissue samples were dissected 5–75 min after leptin/vehicle treatment and subjected to western blot (**c**) and densitometric analyses (**d, e**). Data in **b, d** and **e** are means ± SEM. Differences were analyzed by a two-tailed Student’s *t*-test. **p < 0.01, ***p < 0.001 between ME and MBH samples. (Each data point consists of samples from 2 animals, which were pooled due to detection limitations. *n* = 4 per group for **b** and *n* = 2–3 for **d**, **e**). ME median eminence, MBH mediobasal hypothalamus. The scale bar in **a** is 200 µm

Due to the conflicting nature of the literature regarding leptin transport and uptake in obese animals [12, 17, 25, 26], we aimed to confirm our findings using an additional, more quantitative method. A recent study by Balland and colleagues describes a dissection method to separate the ME from the MBH, thus excluding circulating signals and allowing the measurement of proteins found only within the MBH and not within the ME [17]. By adapting this method we were able to isolate the ME and MBH (Fig. 3a) from freshly extracted brains. We confirmed the accuracy and purity of the dissection by analyzing mRNA levels of two ME residing tanycyte genes using qPCR (Fig. 3b). Both tanycycte genes fibroblast growth factor 10 (FGF10) and dopamine- and cAMP-regulated phosphoprotein (Darpp32) were highly enriched in ME tissue samples and only minimally expressed in MBH samples (Fig. 3b). Having established MBH dissection, we wished to define the time point at which leptin activity is at its peak within the MBH. We measured pSTAT3 in ME & MBH samples 5, 15, 30, 45 and 75 min after leptin injection (i.p., 5 mg kg^−1^) in chow fed mice (Fig. 3c). In ME samples there was no change in pSTAT3 levels (Fig. 3d). However, we found a time-dependent increase in pSTAT3 levels in the MBH, which peaked 45 min after leptin injection and began to decrease thereafter (Fig. 3e). For future experiments, we selected the 45 min time point to allow maximum leptin transport into the MBH.

### Profound weight loss induces upregulation of LepR in the CP and increased leptin accumulation in the MBH

**Fig. 4:**
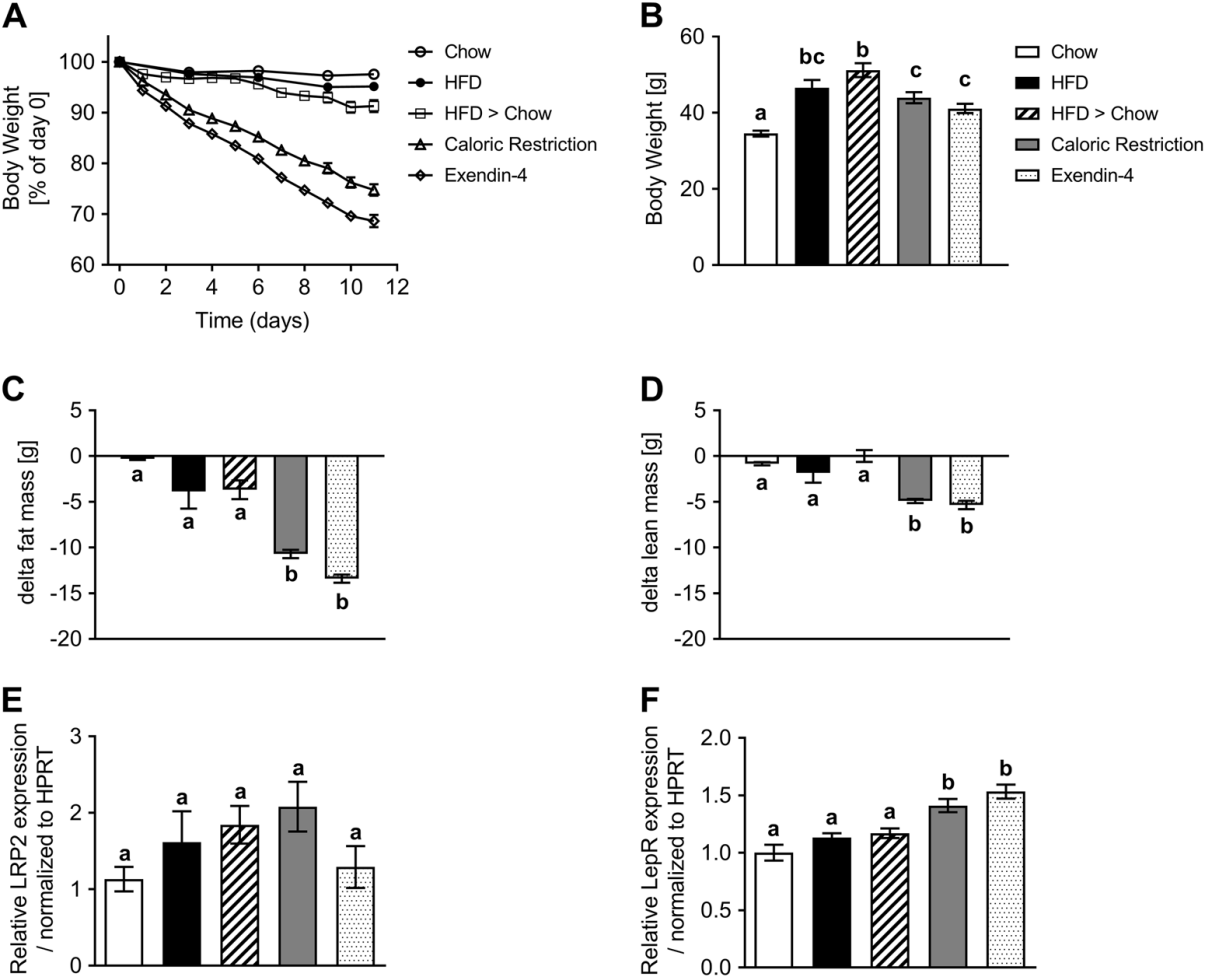
Weight loss by CR or EX4 treatment drives upregulation of leptin receptor mRNA in the choroid plexus. **a** Effects of obesity and weight loss on the transport of leptin into the MBH were recorded in age-matched mice exposed to at least 20 weeks of chow or HFD (Chow, HFD) and additional groups of age-matched and HFD-fed DIO mice after a diet intervention (HFD > Chow; CR) or pharmacological treatment (EX4). Body weights were recorded daily for all groups (chow and HFD every 3 days) for 11 days during the intervention. **b**–**d** Final day body weights for all animals (**b**) as well as changes in fat and lean mass (**c**, **d**). **e**, **f** On day 11, the choroid plexus was isolated from all mice and expression levels of LRP2 and LepR were measured. All data are means ± SEM. Groups with the same character indicate groups that are not significantly different from each other. Groups with different characters are significantly different from each other as determined by One-Way or Two-Way ANOVA with Tukey’s post-hoc testing (*n* = 11–13 for **a**–**d** and *n* = 9–10 for **e**, **f**. HFD high fat diet, H > C diet switch, CR calorie restriction, EX4 exendin-4)

To gain further insight into how leptin resistance may affect leptin transport, we designed a diet intervention study. Control mice fed chow or HFD maintained their bodyweight throughout the study period and the HFD group was significantly heavier (Fig. 4a, b). Additional groups of HFD-fed mice lost weight due to a simple diet switch to chow (HFD > Chow), due to a switch to chow with calorie restriction (CR), or due to a switch to chow plus daily treatment with leptin resensitizing glucagon-like peptide 1 (Glp1) agonist exendin-4 (EX4) [22]. The HFD > Chow group lost 9% BW over the 11 day study period (Fig. 4a), similar to that seen by others [27]. Both weight loss intervention groups CR and EX4 lost significant amounts of body weight (Fig. 4b), totaling 26% and 32%, respectively (Fig. 4a). The majority of lost weight derived from a decrease in fat mass (Fig. 4c), accompanied by significant losses in lean mass (Fig. 4d).

Two possible transporters responsible for the movement of leptin from the CP to the CSF are low density lipoprotein-related protein 2 (LRP2) [28] or the short form of the leptin receptor (LepRa) [18, 19]. We extracted the CP from mice in the diet intervention study and examined mRNA levels of LRP2 and LepR (Fig. 4e–f). LRP2 expression levels were much lower when compared to LepR levels (average CP values of 33 for LRP2 vs. 25 for LepR) and unchanged between groups (Fig. 4e). No changes in LepR expression were seen between chow and HFD groups. Interestingly, LepR was significantly upregulated in both weight loss groups CR and EX4, compared to all groups that did not undergo profound weight loss (Fig. 4f).

**Fig. 5:**
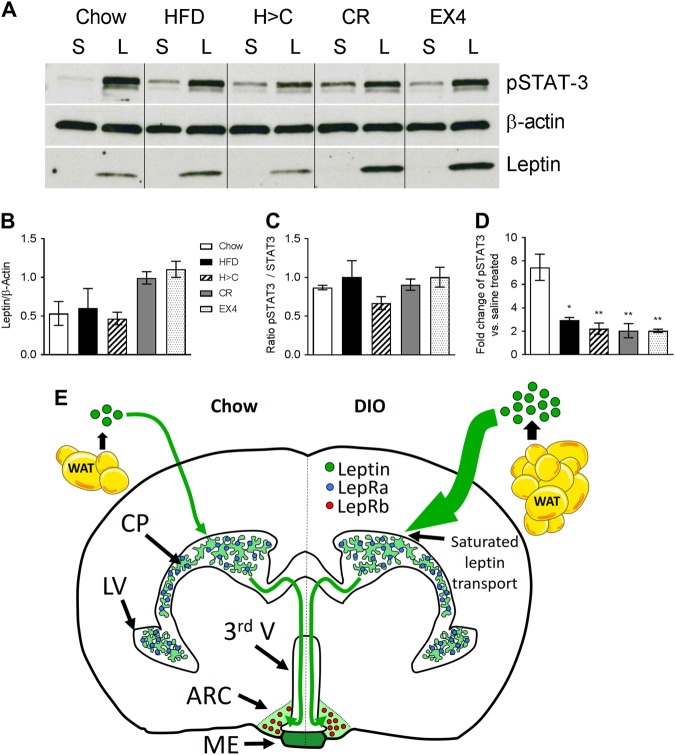
Profound weight loss is associated with increased leptin transport into the MBH. **a**–**d** Leptin levels in the MBH 45 min after vehicle (saline, S) or leptin (L) injections (i.p., 5 mg kg^−1^) in age-matched mice exposed to chow, HFD, H > C, CR or EX4 treatment. Mice were dissected and MBH samples subjected to western blotting (**a**) and densitometric analyses (**b**–**d**) for leptin or leptin sensitivity marker pSTAT3. **e** Schematic representation of the proposed route of leptin transport to the MBH in chow and DIO mice. Each diet group consists of 6 animals, however samples were pooled due to detection limitations, resulting in *n* = 3 replicates for statistical analyses. All data are means ± SEM. Significance was determined using one-way ANOVA. HFD high fat diet, H > C diet switch, CR calorie restriction, EX4 exendin-4. Significance is depicted as *p < 0.05, **p < 0.01

We next aimed to assess the impact of weight loss and increased CP LepR expression on the levels of leptin as well as leptin signaling marker pSTAT3 in microdissected MBH tissue (Fig. 5a–d). Western blotting revealed no differences in pSTAT3 or leptin levels in the MBH between chow and HFD animals (Fig. 5a, d), which corroborates the lack of difference observed in our LSFM analyses. Leptin caused a 7 fold increase in pSTAT3 levels, which was significantly reduced in all other diet groups (Fig. 5d). Intriguingly though, we see elevated leptin levels in the MBH of both weight loss intervention groups CR and EX4, when compared to the other 3 groups (Fig. 5a, b), which correlates with the increased LepR expression seen in the CP of these animals (Fig. 4f).

## Discussion

We describe here a novel method of visualizing the transport of leptin across the BBB in mice. By using fluorescently labeled leptin in combination with tissue clearing and light-sheet fluorescence microscopy, we display highest leptin accumulation in the choroid plexus of the ventricular system followed by circumventricular organs such as the ME or SFO. Notably, we found no differences in leptin accumulation between lean and obese mice in the ME, MBH or CP. Comparable leptin protein levels in microdissected ME and MBH tissues of lean and obese mice, quantified via western blotting, corroborated the results of our microscopy analyses. Overall, we found that DIO does not prevent the accumulation of leptin in brain areas important for metabolic control. Moreover, we revealed that weight loss intervention, triggered either by calorie restriction or pharmacological treatment with exendin-4, led to an increase in leptin receptor expression in the CP as well as elevated leptin protein levels in the MBH.

BBB transport of peptides has traditionally been assessed by radioactive tracer studies [12], which combine high sensitivity with excellent quantification capabilities. However, high costs and difficulties in creating the stable peptide tracer conjugates restrict their use to few laboratories specialized in such studies. Our novel method may be a useful tool to complement or even replace radioactive tracer studies. Foremost, our method does not require radiation and the predicaments associated with it. The kit-based chemistry reaction for the coupling of available fluorescent dyes can be readily adapted to specific peptide hormones. Here, by using infrared fluorescent dyes, researchers can ensure that autofluorescence does not impede the imaging. Next, if labeling with the fluorescent dye is successful and the labeled peptide remains bioactive, modern imaging techniques can be applied to visualize the transport route of fluorescent peptides into the brain. Our post mortem imaging approaches were based on LSFM as well as confocal microscopy to combine whole brain 3D imaging with subsequent high resolution imaging in brain slices. It also seems feasible to study the in vivo uptake of fluorescently labeled peptide into mouse tissues by using alternative biofluorescence imaging techniques. A similar approach, although post mortem, was recently applied to profile the whole body bio-distribution of a fluorescently labeled glucagon-T3 hybride peptide in mice [29].

Our 3D imaging revealed leptin accumulation predominantly in the CP of the ventricular system, an ependymal secretory organ designed to transfer molecules from the blood to the CSF. The CP has been linked to leptin transport into the brain [5, 18, 21, 30], driven by the short form of the leptin receptor that is highly expressed in the CP [19, 31, 32]. Recent work further revealed diminished delivery of leptin across the BBB and increased DIO when all forms of LepR were partially knocked down in BBB-specific endothelial cells of the CP and related areas of mice [18]. A lack of leptin transport into the MBH was further reported for mice with a genetic mutation in the leptin receptor gene (db/db), compared to WT controls [17]. This is however unexpected, as the db/db mutation affects only the signal transducing LepRb isoform. Db/db mice retain a fully functioning LepRa, and have indeed been reported to transport leptin across the BBB via a saturable mechanism [33].

These reports collectively suggest that LepRa expression in the CP is a critical regulator of BBB transport of leptin. Our data contribute to this concept by showing a clear association between elevated LepR levels in the CP and elevated leptin protein levels in the MBH of mice subjected to profound weight loss via CR or EX4. Notably, we observe very low CP expression levels of Lrp2, a transport protein previously linked with leptin BBB transport in CP epithelium [28]. Overall, our results support a model whereby leptin bio-distribution in the brain is predominantly facilitated by the CP and LepR-driven transfer from blood into the CSF.

The degree and more importantly the type of vasculature present in a particular brain region will play a pivotal part in which blood borne substances are given access to the parenchyma. It has been previously shown that the vasculature of the MBH undergoes vascular remodeling during obesity, which directly influences the accessibility to circulating substances [34, 35]. Langlet and colleagues further showed that during fasting, mice undergo vascular remodeling within the ARC [36]. These changes include an invasion of fenestrated vessels from the ME into the ARC, allowing for an increase in peripheral hormone visibility to the arcuate neurons [36]. Thus in a fasting state, which is exhibited in our CR and EX4 models, it is possible that higher levels of circulating leptin could gain access to the ARC, which may additionally contribute to the increased transport by the CP, which we describe here.

Several recent studies suggest a prominent role of the median eminence and tanycytes in mediating leptin transport to the MBH [17, 36]. Tanycytes are specialized ependymal cells that form the blood–CSF barrier in circumventricular organs [37]. To avoid free access of blood borne molecules to the rest of the MBH, tanycytes form a border via tight junctions between their processes [38], and wall off the ME from MBH [39]. Modulation of tanycytic tight junctions in the ME was shown to increase leptin transport into the MBH [17]. Moreover, although leptin receptor expression has never been directly shown (largely due to a lacking suitable antibody), leptin receptor mRNA is expressed in tanycyctes. Furthermore, isolated tanycyctes display phosphorylation of STAT3 upon leptin stimulation, indicating the expression of the leptin receptor. These tanycyctes appear to mediate leptin transcytosis to the CSF and from there to the arcuate nucleus [17]. Our LSFM revealed comparable leptin-CW800 accumulation in the ME of lean and DIO mice. Confocal microscopy further revealed that CW650-leptin levels are accumulating in the cytosol of ß2 tanycytes, and here predominantly at the ventricular side. Accordingly, our imaging data are in line with these earlier reports. However, future studies are warranted to fully delineate the relative contributions of the ME and the CP in mediating BBB transport of leptin to the MBH and other LepR-positive brain areas.

In our study, we see no differences in the accumulation of leptin in the ME or MBH between lean and DIO mice. These data are discrepant to a previous report that, using essentially the same western blotting methodology, found severely impaired transport of leptin into the MBH of DIO mice compared to lean controls. Nevertheless, both our and the previous study [17] report increased pSTAT3 in the MBH after leptin injection, which suggests sufficient leptin transport to elicit leptin signaling. Leptin resistance, it would appear then, is unlikely to be the result of blunted leptin transport into the MBH. Consistent with that notion, we observed elevated basal pSTAT3 levels in the MBH of vehicle-treated DIO mice, indicating chronically activated leptin signaling due to high endogenous leptin levels. This resonates with a recent study by Ottaway and colleagues who, by using leptin receptor antagonist administration, demonstrated that endogenous leptin is still functional in DIO mice [25]. Earlier studies further reported a complete lack of physiological responses such as weight loss or food intake in DIO mice subjected to intracerebroventricular injections of exogenous leptin, but a low-level preservation of leptin-induced hypothalamic Stat3 activation [13]. Overall, these data highlight the possibility of a non-transport related leptin resistance.

How can we reconcile the apparent discrepancies between our results and previous reports, mostly based on radioactive tracer studies, which suggest diminished leptin BBB transport in a state of obesity [12, 17]? These earlier studies largely focused on assessing the steady-state kinetics of leptin BBB transport, i.e., the rate of transfer in relation to the concentrations of circulating tracer. Our measurements were made at a fixed point in time when leptin accumulation reached a maximum in the MBH. Accordingly, we did not evaluate the ratio of leptin entering the brain in relation to the levels in circulation. Our data are nonetheless in agreement with a very recent report on unchanged leptin BBB transport kinetics in mice fed chow or two weeks of HFD, which was built upon a novel micro-perfusion technique and the measurement of MBH leptin over a timeframe of 6 h [40].

We acknowledge, as discussed above, that leptin transport appears to be driven by leptin receptor in a saturable manner. Indeed, LepRa expression levels may be the limiting factor for leptin BBB transport rates when endogenous leptin levels are high, or supraphysiological levels of recombinant leptin are administered. However, as shown by our study and outlined in Fig. 5d, even a putative threshold defined by delimited LepRa expression in the CP and impaired leptin BBB transfer kinetics cannot prevent the entry of significant levels of leptin into the CSF and LepR-expressing brain areas such as the MBH.

Here, we demonstrate a novel visualization of leptin distribution in the whole intact mouse brain. The methods described can be readily adapted to accommodate a plethora of other peptide hormones, which may greatly impact the field of hormonal BBB transport. Taken together, we show that despite being leptin resistant, DIO mice retain a functional leptin transport system and display no deficit in leptin accumulation in the MBH or circumventricular organs. Our findings are further solidified by a recent hypothalamic perfusion study, which revealed no changes in leptin transport kinetics [40]. Moreover, emerging evidence suggests persistence of leptin signaling also in DIO mice and obese humans [25, 41]. Lastly, AgRP and POMC neurons in the MBH are also projecting LepR-expressing somato-denritic processes into the ME, where circulating leptin can induce neuronal leptin signaling without the need of crossing the BBB [42, 43]. Accordingly, in light of recent and our own findings, little evidence points towards impaired leptin BBB transfer as underlying cause for leptin resistance. Future research efforts should thus perhaps be shifted away from leptin transport across the BBB and focused on other possible causes of leptin resistance, which, if corrected has the potential to have a large impact on the treatment of obesity.

## Electronic supplementary material

Supplementary Figure 1 and Supplementary Video Legends

Supplemental Video S1

Supplemental Video S2

Supplemental Video S3

Supplemental Video S4

Supplemental Video S5
