# Supplementary material for: Fluorescent blood–brain barrier tracing shows intact leptin transport in obese mice
Source: Int J Obes (Lond). 2018 Oct 3;43(6):1305–18. doi: 10.1038/s41366-018-0221-z (PMC6760579; doi:10.1038/s41366-018-0221-z)
Supplement: Supplementary file 1 — Supplementary Figure 1 and Supplementary Video Legends [file 41366_2018_221_MOESM1_ESM.docx]

**Supplementary Figure and Videos**

**
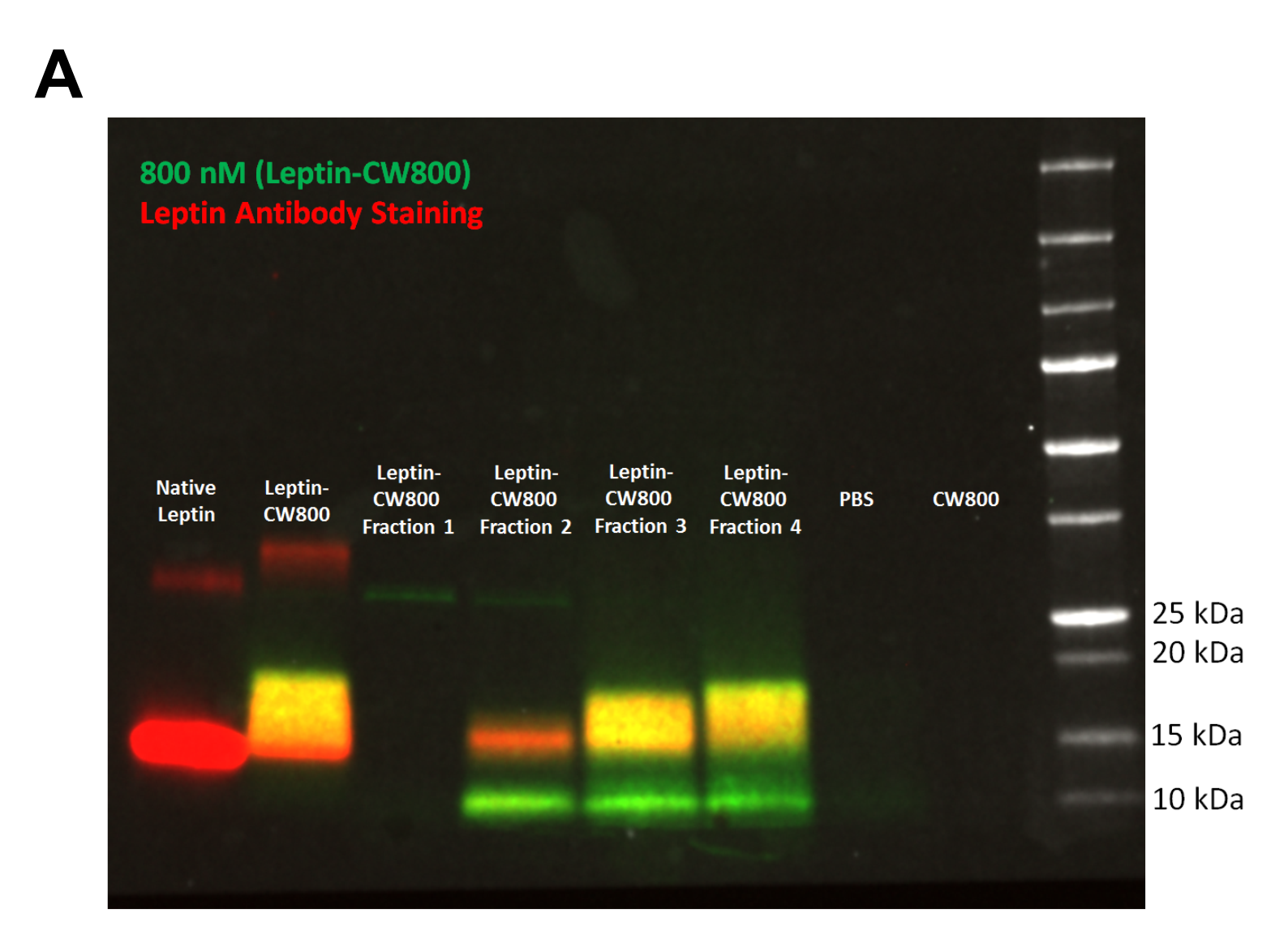
**

**Supplementary Figure 1 | Overexposed image of leptin fractions.**

Leptin, leptin-CW800, fractions F1-F4, PBS and CW800 alone were subjected to SDS-Page and western blotting. Leptin bands were detected by either immunolabeling for a leptin antibody (red) or infrared fluorescence (green). The leptin band is purposefully overexposed as to show the weak bands within the fractions F2-F4.

**Supplementary Video S1 | Coronal 3D reconstruction of leptin-CW800 distribution in the hypothalamus and CP.**

3D-reconstruction of light-sheet fluorescence microscopy (LSFM) images from the midsections of brains of chow-fed lean (left side) and HFD-fed DIO mice (right side) injected with leptin-CW800 (i.p., 5 mg·kg-1) and lectin-647 (i.v., 250 µg). The video, rendered from coronal LSFM scans from bregma 0.34 mm to -3.38 mm, reveals leptin accumulation in the ME and CP of the lateral ventricle (Lectin-647 shown in red, leptin-CW800 shown in green).

**Supplementary Video S2 | Sagittal 3D reconstruction of leptin-CW800 distribution in a whole mouse brain.**

3D-reconstruction of light-sheet fluorescence microscopy (LSFM) images from the brain of a chow-fed lean mouse injected with leptin-CW800 (i.p., 5 mg·kg-1) and lectin-647 (i.v., 250 µg). The video, rendered from sagittal LSFM scans, reveals leptin accumulation in the ME and CP (Lectin-647 shown in red, leptin-CW800 shown in green).

**Supplementary Video S3 | Coronal 3D reconstruction of leptin-CW800 distribution in the hypothalamus and CP of a chow-fed lean mouse.**

3D-reconstruction of light-sheet fluorescence microscopy (LSFM) images from the midsections of brains of a chow-fed lean mouse injected with leptin-CW800 (i.p., 5 mg·kg-1) and lectin-647 (i.v., 250 µg). The video, rendered from coronal LSFM scans from bregma 0.34 mm to -3.38 mm, reveals leptin accumulation in the ME and CP (Lectin-647 shown in red, leptin-CW800 shown in green).

**Supplementary Video S4 | Coronal 3D reconstruction of leptin-CW800 distribution in the hypothalamus and CP of a HFD-fed DIO mouse.**

3D-reconstruction of light-sheet fluorescence microscopy (LSFM) images from the midsections of brains of a HFD-fed DIO mouse injected with leptin-CW800 (i.p., 5 mg·kg-1) and lectin-647 (i.v., 250 µg). The video, rendered from coronal LSFM scans from bregma 0.34 mm to -3.38 mm, reveals leptin accumulation in the ME and CP (Lectin-647 shown in red, leptin-CW800 shown in green).

**Supplementary Video S5 | Coronal 3D reconstruction of leptin-CW800 distribution in the choroid plexus.**

3D-reconstruction of light-sheet fluorescence microscopy (LSFM) images from choroid plexus of a chow-fed lean mouse injected with leptin-CW800 (i.p., 5 mg·kg-1) and lectin-647 (i.v., 250 µg). The video, rendered from coronal LSFM scans from bregma -2.15 mm to -2.35 mm, reveals leptin accumulation as well as uptake into the CP. Leptin-CW800 in surrounding microvasculature is confined to the inside of the vessels (orange signal), whereas in the CP is also seen outside the vessels (green/yellow signal).
